# Effects of Adjunct Low-Dose Vitamin D on Relapsing-Remitting Multiple Sclerosis Progression: Preliminary Findings of a Randomized Placebo-Controlled Trial

**DOI:** 10.1155/2012/452541

**Published:** 2012-04-11

**Authors:** Vahid Shaygannejad, Mohsen Janghorbani, Fereshteh Ashtari, Hamed Dehghan

**Affiliations:** ^1^Department of Neurology, Isfahan Neurosciences Research Center, Isfahan University of Medical Sciences, Isfahan 8144503500, Iran; ^2^Department of Epidemiology and Biostatistics, School of Public Health, Isfahan University of Medical Sciences, Isfahan 8144503500, Iran

## Abstract

The aim of this preliminary study was to evaluate the effect of low-dose oral vitamin D in combination with current disease-modifying therapy on the prevention of progression of relapsing-remitting multiple sclerosis (RRMS). A phase II double-blind placebo-controlled randomized clinical trial conducted between October 2007 and October 2008 included 50 patients with confirmed RRMS aged 25 to 57 years and normal serum 25-hydroxyvitamin D. They were randomly allocated to receive 12 months of treatment with either escalating calcitriol doses up to 0.5 *μ*g/day or placebo combined with disease-modifying therapy. Response to treatment was assessed at eight-week intervals. In both groups, the mean relapse rate decreased significantly (*P* < 0.001). In the 25 patients treated with placebo, the mean (SD) Expanded Disability Status Scale (EDSS) increased from 1.70 (1.21) at baseline to 1.94 (1.41) at the end of study period (*P* < 0.01). Average EDSS and relapse rate at the end of trial did not differ between groups. Adding low-dose vitamin D to routine disease-modifying therapy had no significant effect on the EDSS score or relapse rate. A larger phase III multicenter study of vitamin D in RRMS is warranted to more assess the efficacy of this intervention.

## 1. Introduction

Multiple sclerosis (MS) is an inflammatory, demyelinating, immune-mediated, and debilitating disease that affects young adults and is often associated with significant disability and impaired quality of life [1, 2]. Disability and high morbidity are common features of the disease [3]; symptom management must therefore be a long-term consideration, as disease-modifying therapies only slow the progression of the disease [4]. Despite extensive research to develop effective pharmacological treatments to alleviate exacerbation and chronic neurological damage in MS, current available drugs have limited efficacy and considerable adverse effects [5–7].

Vitamin D is a lipid-soluble vitamin synthesized by conversion of 7-dehydrocholesterol to vitamin D in the skin. Vitamin D is a secosteroid hormone known to play an important role in bone formation and mineral homeostatis. Recent studies have also suggested that vitamin D affects immune and central nervous system (CNS) development and function and has strong immune-regulatory capacity [8–11]. Some *in vitro* and animal studies suggest that vitamin D supplementation reduced inflammatory infiltration in the CNS by suppressing function of antigen-presenting cells [12]. Mice treated with dietary 1, 25-dihydroxyvitamin D_3_ prior to induction of experimental autoimmune encephalomyelitis (EAE), an animal model for MS, did not develop symptoms of EAE, compared with 100% incidence in the control group [12]. In mice with EAE, 1, 25-dihydroxyvitamin D_3_ injections followed by dietary supplementation halted disease progression. The effects of vitamin D were reversible, as evidenced by EAE progression after discontinuation of the supplement. Epidemiological evidence from observational studies also has suggested that vitamin D may help prevent MS, reduce exacerbations, and may be useful addition to standard MS therapy [13]. These findings suggest that the active form of vitamin D may be effective in treating patients with MS.

Whether vitamin D is effective in human MS is not known. There are, however, few small open-label or noncontrolled clinical trials relating vitamin D to MS progression [14–18]. We therefore examined the therapeutic effect of low-dose vitamin D on the prevention of progression of MS. Because vitamin D is able to effectively suppress acute EAE, which has many similarities to MS [11, 12], has low or absent toxicity, and has been safely used in humans orally; even modest therapeutic effectiveness of this drug would be useful in the treatment of MS.

In the present exploratory phase II trial, we compared the effects of low-dose vitamin D and a placebo in combination with current disease-modifying therapy in terms of their safety, tolerability, and relative efficacy in preventing the progression of relapsing-remitting multiple sclerosis (RRMS).

## 2. Patients and Methods

### 2.1. Patients

A total of 50 consecutive patients with RRMS were recruited from neurology outpatient clinics of Isfahan University of Medical Sciences and the Isfahan Multiple Sclerosis Society between October 2007 and October 2008, with the last patient completing the 12 month trial in March 2009. All of the cases had a magnetic-resonance-imaging- (MRI-) supported diagnosis. Entry criteria were either sex, age between 15 and 60 years with a MRI, clinical or laboratory-supported diagnosis of definite RRMS [19] mean (standard deviation (SD)) duration 4.3 (2.2) years, range 1–12 years, stable neurological functioning for at least one month prior to study entry, and an EDSS [19] score ≤6, serum 25-hydroxyvitamin D level >40 ng/mL [20] and a willingness to continue current medications for the duration of the study. Assessments of serum 25-hydroxyvitamin D level were carried out routinely as part of the clinical management of MS and used to detect vitamin D insufficiency. Exclusion criteria were evidence of substantial abnormalities in neurological, psychiatric, cardiac, endocrinological, hematologic, hepatic, renal, or metabolic functions, use of digitalis, vitamin D supplement, any condition predisposing to hypercalcemia, nephrolithiasis, renal insufficiency and pregnancy as determined by history, physical examination, and screening blood tests. Patients with secondary-progressive and primary-progressive MS were excluded to ensure that the treatment group was homogeneous in terms of course of disease and possibly also in disease mechanisms. Tenets of the current version of the Declaration of Helsinki were followed, institutional ethical committee approval was granted, and the nature of the trial was explained to the participants. After a detailed discussion with the neurologist, patients made a final decision, and each participant provided written informed consent. This trial was registered with Iranian Registry of Clinical Trials (ID IRCT201104166202N1).

### 2.2. Randomization Scheme

**Figure 1:**
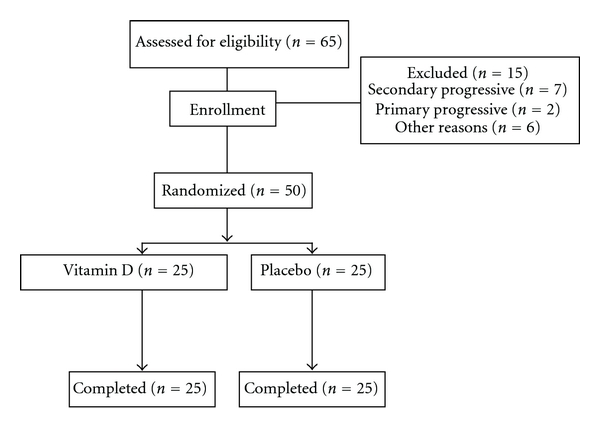
Design of the trial to compare oral vitamin D (0.5 *μ*g/day) versus placebo in patients with relapsing-remitting multiple sclerosis.

A total of 65 patients were eligible for study. Fifteen patients were excluded because their type of MS was not RRMS, they refused entry, or they did not meet the inclusion criteria. Fifty patients (6 (12.0%) men, 44 (88.0%) women) completed the study without interruption and were assigned randomly to one of the two self-administer treatment groups. Patients were randomized according to a preexisting list produced by a computer program. All patients continued on their own RRMS treatment regimen. The first treatment group received 0.25 *μ*g adjunct calcitriol (a metabolically active form of vitamin D (1, 25-dehdroxyvitamin D_3_) (trade name Zavitrol, Zahravi Pharm. Co. Tabriz, Iran) per day and increased to 0.5 *μ*g/day after 2 weeks and continued for 12 months. The second group received placebo for 12 months. Both were administered as capsules twice a day orally before meals. Patients were allowed access to their routine RRMS treatments. Compliance with the study treatment was verified by asking the patients about missed doses and by counting unused sachets. In the month preceding the trial, all patients underwent pretreatment evaluation to record demographic data, complete neurologic and medical history, the finding of physical and neurologic examination, and previous treatment.  Figure 1 illustrates the patient allocation algorithm. In the final sample of participants mean (SD) age was 38.2 (8.4) years (range from 25 to 57 years) and baseline EDSS score ranged from 1.0 to 5.5.

### 2.3. Patient Evaluation

The trial was double-blinded in that both patient and physician who assessed the outcome were unaware of the type of treatment each patient received. Masking of the active and placebo treatments was preserved by creating treatments that looked identical. The hospital pharmacist was informed of all randomization assignments and was responsible for labeling the study drug and maintaining a master list linking participants and their treatment assignments. Participants were evaluated by a qualified neurologist at baseline, 2, 4, 6, 8, 10, and 12 months after the start of the therapy to evaluate the development of side effects of the medications, compliance, and disease activity (EDSS and evaluation of relapse occurrence). Brain MRI with gadolinium was carried out at baseline and 12 months. All patients were evaluated by the same physician (HD), who did not know which patients had received which treatment.

The number of relapses, the proportion of patients free from relapses, the EDSS, and other medical events were recorded at each visit. Acute relapse was defined as the appearance of a new neurological symptom or severe deterioration in a preexisting symptom that lasted for at least 24 h in the absence of fever/infection and caused an increase of at least 1 point in EDSS [21]. The primary outcome measure was deterioration from baseline to 12 months after receiving vitamin D or placebo as measured by the EDSS. Mean changes in relapse rate were also calculated for both groups.

### 2.4. Statistical Analysis

The study was powered (80%) to detect (with a two-sided alpha of 0.05) a mean difference in EDSS score from baseline of 0 point. Statistical analysis was based on the intention-to-treat principle. The results for the groups that received vitamin D or placebo were compared with Student's *t*-test for independent samples and analysis of variance with repeated measures over time; the results at baseline and after 12-months within each group were compared with paired Student's *t*-tests. We used the chi-square or Fisher's exact test to compare proportions. The results are expressed as the mean (SD), and *P* < 0.05 was considered statistically significant. All statistical tests were two-sided. Analyses were done using SPSS for Windows (SPSS Inc., Chicago, IL, USA). There was no interim analysis of treatment effects.

## 3. Results

Fifty patients who met the entry criteria were enrolled in the study. Patient compliance with treatment was good. All 50 patients who completed treatment were available for followup at 12 months. The two treatment groups were generally well matched at baseline with regard to age, gender, duration of RRMS, EDSS, number of relapse-free years prior to the trial, and other MS characteristics. Most participants (86.0%) had received interferon beta, statins (10.0%), or immunosuppressive drugs (4.0%) with no significant differences between groups. Mean (SD) age in the vitamin D and placebo groups was 38.6 (8.4) and 37.9 (7.9) years, respectively. Mean (SD) EDSS at the start of treatment was 1.6 (0.7) in the vitamin D group and 1.7 (1.2) in the placebo group (Table 1). Two patients had two attacks during the preceding year, and the reminders had one attack.

Vitamin D treatment was tolerated well and most of the adverse events reported were mild in severity. The most common side effects of vitamin D were constipation (*n* = 6), dyspepsia (*n* = 6), fatigue (*n* = 4), and headache (*n* = 2). The most common side effects of placebo were also constipation (*n* = 4), dyspepsia (*n* = 2), fatigue (*n* = 5), and headache (*n* = 1). There were no substantial differences between vitamin D and placebo groups in frequency or pattern of events. There were no instances of urinary dysfunction or symptomatic nephrolithiasis.

Changes in mean EDSS and number of relapses before and after receiving vitamin D or placebo are shown in Table 2. In placebo groups, the average EDSS score increased significantly. The average increase from baseline was 0.24 point (95% CI, 0.08–0.40). In patients treated with vitamin D, mean EDSS score did not change. In both groups, the mean relapse rate decreased significantly. Of the 25 patients treated with vitamin D, the mean (SD) relapse rate decreased from 1.04 (0.20) at baseline to 0.32 (0.48) at the end of study period (*P* < 0.001). In the 25 patients treated with placebo, the mean (SD) relapse rate decreased from 1.04 (0.20) at baseline to 0.40 (0.58) at the end of study period (*P* < 0.001). After 12-months, 17 (34.0%) relapses had occurred; 8 (32.0%) in the vitamin D and 9 (36.0%) in the placebo group. After 12 months, 33 of 50 patients (66.0%) remained relapse-free; 17 (68.0%) in the vitamin D and 16 (64.0%) in the placebo group. The odds ratio was 1.06 (95% CI, 0.71–1.58), indicating there is no evidence of an effect on the odds of remaining relapse-free during the first 12 months of therapy in patients who received vitamin D compared to those who received the placebo.

The overall cross-tab analysis revealed no significance differences in the EDSS or number of relapses at the end of the study period between the vitamin D and placebo groups (Table 3).

**Figure 2:**
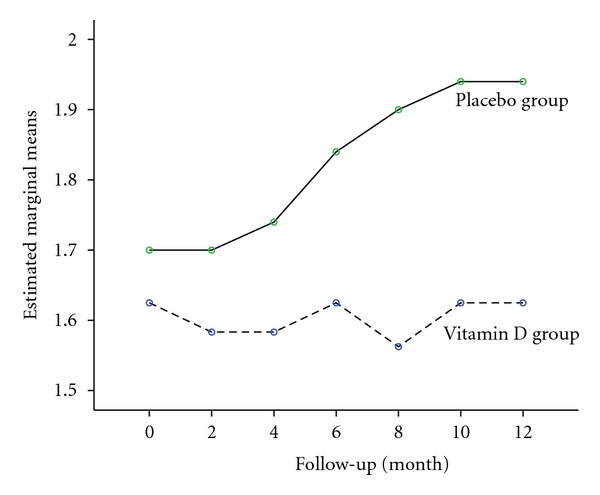
Estimated mean changes in Expanded Disability Status Scale after 2, 4, 6, 8, 10, 12 months of followup.

The overall analysis of repeated measures ANOVA revealed no significance differences in the EDSS at the end of trial between vitamin D and placebo groups (*P* > 0.05) (Table 4).  Figure 2 shows the estimated mean changes in EDSS score after 2, 4, 6, 8, 10, 12 months of followup in two groups. Those receiving vitamin D showed no changes but those receiving placebo showed progressive neurologic deterioration.

## 4. Discussion

In this exploratory phase II study we found no significant difference in relapse rate or change in EDSS between participants who took the placebo versus those who received adjunct low-dose oral vitamin D during 12 months. No unusual or unexpected safety risks were found with vitamin D therapy in our study population with RRMS. The spectrum of most frequent adverse events is similar to that in previous studies of vitamin D treatment for MS, with gastrointestinal side effects being most common. Previous studies have shown that vitamin D is fairly safe [14], especially in terms of its effects on the gastrointestinal tract [14]. We did not find any significant differences between the active drug and the placebo in safety, and there were no distinct patterns in adverse events.

The efficacy of vitamin D for treatment of MS has been examined in noncontrolled trials with variable results. The effect of calcium, magnesium, and vitamin D supplementation on relapse rate was evaluated in 16 young patients with MS [15]. Participants received magnesium 10 mg/kg, calcium 16 mg/kg, and cod liver oil 20 g supplying 5000 IU of vitamin D per day. The primary endpoint of the trial was the actual number of exacerbations compared with the expected number. There was a significant reduction in the actual number of exacerbations compared with the expected number. The authors concluded that calcium, magnesium, and vitamin D supplementation reduced the number of exacerbations experienced by patients with MS. Vitamin D was coadministered with calcium and magnesium, preventing its actual effectiveness. In addition, the source of vitamin D used in this study, cod liver oil, also contains fatty acids, which may have contributed to the positive results. An open-label study evaluated 1, 25-hydroxyvitamin D3 (calcitriol) treatment in 15 patients with RRMS [16]. Oral calcitriol was started at 0.5 *μ*g daily and increased by 0.5 *μ*g/day every 2 weeks until the target dosage of 2.5 *μ*g/day was reached. The authors suggested that calcitriol therapy was unlikely to aggravate symptoms of MS. Another open-label trial of alfacalcidol (activated vitamin D) was conducted in 5 patients with MS [17]. The patients had RRMS, with a relatively low exacerbation rate and received alfacalcidol 1.5 *μ*g/day for 6 months. Three patients remained stable, 1 had improvement in neurologic status, and 1 developed an acute relapse. No adverse effects were reported with use of alfacalcidol. Another pilot study enrolled 11 patients with RRMS to determine the safety and efficacy of 19-nor-1, 25-dihydroxyvitamin D2, a vitamin D analog [18]. Patients received 6 monthly MRI scans prior to starting treatment. After 19-nor-1, 25-dihydroxyvitamin D2 was titrated to the maximally tolerated dose (average 4 *μ*g), participants received an additional 6 monthly MRI scans. While no adverse effects were reported, no significant changes were seen in clinical symptoms or MRI lesions.

In summary, these four studies involved a total of 47 patients, varying in gender, age, dose, type of vitamin D used, type of MS, and follow-up time. Interpretation of data is complicated by the demographic variability in the patients, by the methodological problems related to the outcome measures in these trials, and by their subsequent analyses. In addition, statistical analyses did not always deal with the small number of patients and the small effect sizes.

The efficacy of vitamin D for treatment of MS has been examined in an open-label randomized controlled trial conducted over 52 weeks, which treated 25 patients with escalating doses of vitamin D compared with control [14]. The trail provided some evidence of the potential benefit of the intervention on several outcomes, that is, the annualized relapse rate, EDSS score, and suppression of T-cell proliferation and illustrated a measure of comparative safety in the relative absence of any adverse events or of high serum calcium level over the study period. Recently, the effect of high-dose vitamin D2 (6000 IU/day) compared with low-dose (1000 IU/day) supplementation was evaluated in 23 patients with RRMS [22]. The primary endpoint of the trial was cumulative number of new gadolinium-enhancing lesions and change in total volume of T2 lesions. Secondary endpoints were EDSS and relapse rate. They did not find therapeutic advantages in RRMS for high-dose vitamin D2 compared with low-dose vitamin D2 supplementation. The high-dose group actually had significantly more relapses and worse disability than the low-dose group at the end of the study. Thus in this study, there seemed to be no advantage from high-dose vitamin D supplementation in patients with MS compared to supplementation for adequate vitamin D levels. These studies were too small to make a definitive conclusion about the vitamin's efficacy. To the best of our knowledge, no other studies are available comparing low-dose vitamin D with placebo, and this is the first randomized clinical trial to compare the effect of adjunct low-dose vitamin D in combination with current disease-modifying therapy in preventing the progression of RRMS.

The precise mechanism of action of vitamin D in RRMS has not been fully elucidated yet. Multiple sclerosis has been identified as Th1-mediated autoimmune diseases [23], but it is Th2-mediated responses which have beneficial effects on the severity and progression of the disease [24, 25]. Response mediated by Th2 represents one of the major mechanisms underlying Ag-specific immune tolerance induction [26–29]. Vitamin D has anti-inflammatory action *in vitro*, including enhanced Th2 and decreased Th1 cytokine production, dendritic cell effects, and enhanced macrophage phagocytosis [30, 31]. Vitamin D has been shown to effectively suppress T cell and antigen-presenting cell activation [30, 31]. It is also known to reduce inflammatory infiltration into the CNS in animal model [12]. More importantly, it has been shown to be safe for use in human [14, 16]. *Ex vivo* and *in vitro* studies showed that vitamin D induced blockade of Th1 and upregulation of Th2 response [12]. These results suggest vitamin D is able to induce the Th2 response and inhibit acute autoimmune attack on the CNS.

Albeit, this study is one of the largest placebo-controlled trials to date of effect of oral vitamin D in combination with current disease-modifying therapy on the prevention of progression of RRMS; the strongest limitation of it is the small number of patients included, and it seems unlikely that small effect can be ruled out. The efficacy should therefore be tested in a larger sample. The present results clearly need to be replicated and extended across multiple centers and investigators. The possible explanation for the discrepancies between these results and those of previous studies might be related to some methodological differences, such as patient selection, the examination of patients at different stages in the natural history of MS, and vitamin D type and dosage. We used 0.5 *μ*g calcitriol per day for 12-months, while Burton et al. [14] used up to 40,000 IU/day vitamin D for over 28 weeks, Wingerchuk et al. [16] used oral calcitriol 2.5 *μ*g/day for 48 weeks, Goldberg et al. [15] used up to 5,000 IU of vitamin D per day for one to two years, and Achiron et al. [17] used alfacalcidol 1.5 *μ*g/day for 6 months. Although substantial evidence supports the safety of even large dose of vitamin D, such evidence is based on studies of limited size and duration. The best level of vitamin D for health is uncertain. Many experts believe that blood levels of vitamin D above 30 ng/mL are adequate [32]. Most found that patients with MS appeared to benefit from having adequate vitamin D levels compared to insufficiency or deficiency. A few studies also suggested that levels higher than 30 ng/mL may further help protect patients with MS [32]. Therefore, our study suggests that the dose of 0.5 *μ*g/day vitamin D in patients with sufficient serum 25-hydroxyvitamin D level may be considered relatively safe.

In conclusion, this exploratory phase II comparative trial of low-dose vitamin D supplementation and placebo showed that adding vitamin D to routine disease-modifying therapy had no significant effect on the EDSS score or relapse rate. Further studies with larger sample and longer followup are needed.

## Figures and Tables

**Table 1:** Characteristics of patients with relapsing-remitting multiple sclerosis who received low-dose vitamin D (0.5 *μ*g/day) or placebo at baseline.

Characteristics	Treatment group at baseline		Differences (95% CI)
	Vitamin D (*n* = 25) Mean (SD)	Placebo (*n* = 25) Mean (SD)	
Age (years)	38.6 (8.4)	37.9 (7.9)	0.7 (−3.9, 5.3)
Duration of multiple sclerosis (years)	4.5 (2.7)	4.1 (1.7)	0.4 (−0.9, 1.7)
EDSS at baseline	1.6 (0.7)	1.7 (1.2)	−0.1 (−0.7, 0.5)
Relapses in previous year	1.04 (0.2)	1.04 (0.2)	0.0 (−0.1, 0.1)

	No. (%)	No. (%)	

Gender			
Men	3 (12.0)	3 (12.0)	0.0 (−18.0, 18.0)
Women	22 (88.0)	22 (88.0)	—
Concomitant medications			
Interferon beta	22 (88.0)	21 (84.0)	4.0 (−15.2, 23.2)
Statin	2 (8.0)	3 (12.0)	−4.0 (−20.6, 12.6)
Immunosuppressive drug	1 (4.0)	1 (4.0)	0.2 (−10.9, 10.9)
EDSS at baseline			
≤1.5	15 (60.0)	18 (72.0)	−12.0 (−38.0, 14.0)
2.0–2.5	8 (32.0)	4 (16.0)	16.0 (−7.3, 39.3)
≥3.0	2 (8.0)	3 (12.0)	−4.0 (−20.6, 12.6)
Relapses in previous year			
1	24 (96.0)	24 (96.0)	0.0 (−10.9, 10.9)
2	1 (4.0)	1 (4.0)	0.0 (−10.9, 10.9)

CI: confidence interval, EDSS: expanded disability status scale.

**Table 2:** Comparison of Expanded Disability Status Scale (EDSS) and relapses in 50 patients with relapsing-remitting multiple sclerosis before and 12 months after treatment with low-dose vitamin D and placebo.

Treatment group	Number	Baseline Mean (SD)	12 months after therapy Mean (SD)	Differences (95% CI)
	EDSS			
Vitamin D	25	1.63 (0.73)	1.63 (0.70)	0.0 (−0.15, 0.15)
Placebo	25	1.70 (1.21)	1.94 (1.41)	−0.24 (−0.40, −0.08)*

	Relapses			
Vitamin D	25	1.04 (0.20)	0.32 (0.48)	0.72 (0.50, 0.94)**
Placebo	25	1.04 (0.20)	0.40 (0.58)	0.64 (0.38, 0.90)**

*P < 0.01, **P < 0.001, CI: confidence interval.

**Table 3:** Comparison of Expanded Disability Status Scale (EDSS) and relapses in 50 patients with relapsing-remitting multiple sclerosis after 12 months of treatment with low-dose vitamin D and placebo.

	Treatment group		Differences (95% CI)
	Vitamin D (*n* = 25) Mean (SD)	Placebo (*n* = 25) Mean (SD)	
EDSS at 12-months	1.63 (0.70)	1.94 (1.41)	−0.31 (−0.94, 0.32)
Relapses at 12-months	0.32 (0.48)	0.40 (0.58)	−0.08 (−0.38, 0.22)

**Table 4:** Comparison of Expanded Disability Status Scale (EDSS) and relapses in 50 patients with relapsing-remitting multiple sclerosis before and 2, 4, 6, 8, 10, 12 months after treatment with low-dose vitamin D and placebo.

Characteristics	Treatment group		Differences (95% CI)
	Vitamin D (*n* = 25) Mean (SD)	Placebo (*n* = 25) Mean (SD)	
	EDSS		
At baseline	1.60 (0.72)	1.70 (1.22)	**−**0.10 (**−**0.67, 0.47)
After 2 months	1.56 (0.70)	1.70 (1.22)	**−**0.14 (**−**0.71, 0.43)
After 4 months	1.56 (0.70)	1.74 (1.23)	**−**0.18 (**−**0.75, 0.39)
After 6 months	1.60 (0.70)	1.84 (1.32)	**−**0.24 (**−**0.84, 0.36)
After 8 months	1.54 (0.66)	1.90 (1.42)	**−**0.36 (**−**0.99, 0.27)
After 10 months	1.63 (0.70)	1.94 (1.41)	**−**0.31 (**−**0.94, 0.32)
After 12 months	1.63 (0.70)	1.94 (1.41)	**−**0.31 (**−**0.94, 0.32)
Difference at 12-months and baseline	0.03 (0.36)	0.24 (0.39)	**−**0.21 (**−**0.45, **−**0.3)*

	Relapses		
At baseline	1.04 (0.20)	1.04 (0.20)	0.00 (**−**0.11, 0.11)
After 12 months	0.32 (0.48)	0.40 (0.58)	**−**0.08 (**−**0.38, 0.22)
Difference at 12-months and baseline	**−**0.72 (0.54)	**−**0.64 (0.64)	**−**0.08 (**−**0.42, 0.26)

*P < 0.05. CI: confidence interval.
